# Evaluation of antimicrobial susceptibility and integron carriage in *Helicobacter pylori *isolates from patients

**Published:** 2016-12

**Authors:** Mehdi Goudarzi, Mohsen Heidary, Mehdi Azad, Maryam Fazeli, Hossein Goudarzi

**Affiliations:** 1 *Department of Microbiology, School of Medicine, Shahid Beheshti University of Medical Sciences, Tehran, Iran*; 2 *Department of Medical Microbiology, Faculty of Medicine, Iran University of Medical Sciences, Tehran, Iran*; 3 *Department of Medical Laboratory Sciences, School of Paramedicine, Qazvin University of Medical Sciences, Qazvin, Iran*; 4 *WHO Collaborating Center for Reference and Research on Rabies, Pasteur Institute of Iran, Tehran, Iran*

**Keywords:** *H. pylori*, integron, Multidrug-Resistant

## Abstract

**Aim:**

The purpose of this study was to determine the antibiotic susceptibility pattern and distribution of integron in *H. pylori *isolates collected from patients referred to private health care centers in Tehran, Iran.

**Background:**

Antibiotic resistance is the main reason for failure of *Helicobacter pylori *therapy. Integrons as genetic reservoirs play main roles in the dissemination of antimicrobial resistance gene.

**Methods:**

During a 12-month cross-sectional study period, 65 *H. pylori *isolates were recovered from 124 biopsy specimens. Isolates were subjected to susceptibility testing using by Epsilometer test according to the European Committee on Antimicrobial Susceptibility Testing (EUCAST) guideline. PCR was used to detect different types of integrons.

**Results:**

Antimicrobial susceptibility testing revealed that 73.8% of isolates were resistant to metronidazole, 43.1% to clarithromycin, 29.2% to tetracycline, 27.7% to amoxicillin, 23.1% to rifampicin and 13.4% to levofloxacin. Frequency of multidrug resistance among

*H. pylori *isolates was 26.1%. The most predominant resistance profiles among our isolates were included resistance to clarithromycin and metronidazole (20%). Class 1 and 2 integrons were detected in 8 (12.3%) and 15 (23.1%) of the isolates, respectively.

**Conclusions:**

The high prevalence of multidrug resistance and frequency of class 2 integron in this survey can be a warning for clinicians. Continuous surveillance is necessary for the development of new treatment protocols to prevent the treatment failures and also further spread of resistant isolates.

## Introduction

*Helicobacter pylori *(*H. pylori*) as one of the most common chronic bacterial infections colonize the stomachs of about 50- 60% of the world’s population. The *H. pylori-*related digestive diseases can range from mild or chronic gastritis to peptic ulcer, gastric lymphoma and gastric cancer and appears to occur in childhood and in most cases remains for all lifetime. Based on the World Health Organization International Agency for Research on Cancer (WHO/IARC), this bacterium is considered in group 1 human carcinogen (1). From the past decades, this bacterial infection is a serious threat to public health in developing countries as well as in developed countries (2).

Although the main routes of infection have not been clarified yet, it is well established that person-to-person contact, oral-oral and fecal-oral routes could be major routes of human infection (3). The prevalence of *H. pylori *infection varies globally in different populations and is associated with a geographic area, socioeconomic factors, personal hygiene and age (1, 3). Proper antibacterial therapy as an effective factor plays crucial role in the eradication of *H. pylori *and thereby reducing the severity of gastric disease symptoms or completes recovery of patients. Combination therapy with a variety of antimicrobial agents such as proton pump inhibitor (PPIs), macrolide and a β-lactam as an eradication regiment could be effective for treatment of *H.pylori *infections (4). Unfortunately, during the past several decades, with the emergence of multi-resistant strains, the successful treatment of *H. pylori *have been a challenge.

Global emergence of *H. pylori *antibiotic resistance not only leads to an increase in economic burden, but it may also cause limited the choice of available therapeutic options for treatment of *H. pylori *infections (2, 5).

Several mechanisms of resistance to antibiotics such as point mutations, redox intracellular potential, pump efflux systems, membrane permeability and penetration of bacterium in the mucous layer of stomach are accurately detailed due to the extreme genetic heterogeneity of *H. pylori *(6). In general, resistance mechanism of *H. pylori *is still a complex problem but mutation in the chromosomal genes or horizontal gene transfers mediated by mobile genetic elements (e.g., plasmids and transposons) are the most probable mechanisms in *H. pylori *(7). Nevertheless, on the basis of the most recent data available from the literature and limited ability of antimicrobial resistance plasmids to replicate in *H. pylori*, there are contradictory results about spreading antibiotic multi-resistance in *H. pylori *strains and the existence of integrons (7, 8).

Integrons include a site-specific recombination system capable of capturing mobile genes that lead to the spread of multi-drug resistance (MDR) particularly in gram- negative pathogens, but, have also been described in gram positive bacteria. They normally are motionless, but, can be transferred through mobile genetic elements, e.g., plasmids and transposons. The core integron structure consists of a primary recombination site (*attI*), integrase gene (*intI*) and a strong promoter gene (9). To date, several classes of integrons have been recognized, but, three classes in particular, classes 1, 2, and 3, have clinical significance. Class 1 integrons are often isolated from gram-negative MDR pathogens. Class 2 integrons with less frequency compare to class 1 reported in gram-negative bacteria. Other classes of integrons were reported rarely (7, 10).

The aims of this study were to evaluate the pattern of antibiotic resistance by micro-broth dilution method, and also, determination of integron frequency in *H. pylori* isolates collected from patients referred to private health care centers in Tehran, Iran.

## Patients and Methods

**Patients and Bacterial Strains**

In this cross-sectional descriptive study from 1 January tubes containing 10 ml Stuart medium (Merck, Germany) and transported to the microbiology laboratory within 4 h of collection. For bacterial identification, homogenized gastric biopsy samples were cultured in Brucella blood agar (Merck, Germany) containing defibrinated sheep blood (5%) and antibiotic supplements (vancomycin 5 mg/L, trimethoprim 5 mg/L, and polymyxin B 0.25 mg/L). The plates were incubated under microaerophilic conditions (5% O2, 10% CO2, 85% N2) at 37°C and high humidity for 3 to 7 days. Organisms were identified as *H. pylori *based on gram stain, culture, oxidase, catalase and rapid urease test. Samples confirmed as *H. pylori *isolates were stored in Brain Heart Infusion broth containing (BHI; Merck, Germany) containing 30% glycerol at -70°C for further studies.

**Antimicrobial susceptibility testing**

*In vitro *susceptibility testing for both *H. pylori *isolates and the reference strain (*H. pylori *ATCC 26695) was performed by Epsilometer test (E-test) with the broth microdilution method. Interpretation for the susceptibility was based on the guidelines of the European Committee on Antimicrobial Susceptibility Testing (EUCAST) (www. eucast.org). The Minimum inhibitory concentration (MIC) values were defined as the lowest antibiotic concentration that completely inhibited visible growth of the tested isolate. The antimicrobial agents used in the present survey include: metronidazole, levofloxacin, clarithromycin, amoxicillin, rifampicin and tetracycline. The MIC was determined by use of the E-test strips (Epsilometer test; Biomeriux, Solna, Sweden). To perform *in vitro *susceptibility testing, Mueller HintonAgar (MHA) with 5% sheep blood were prepared and inoculated with adjusted inoculum suspensions (McFarland no. 4). Then, E-test strip was aseptically placed on to the dried surfaces of each inoculated agar plate and incubated for 72 hours at 37°C. The MIC value was read after 48–72 h of incubation as intersect where the ellipse of growth inhibition intersects the strip. MIC and MIC (MICs that inhibit 50% and 90% of the isolates) were estimated for each antibiotic.

**Extraction of genomic DNA**

DNA was extracted from specimens using a commercially available kit; QIAamp DNA isolation columns (Qiagen, Hilden, Germany). DNA purity was assessed by spectrophotometer.

2014 to the end of December 2014, a total of 65 *H. pylori *The ratio OD 260/ OD280 was calculated and DNAsample within

isolates was recovered from 124 biopsy specimens of patients referred to private health care centers of Tehran (capital city of Iran). At least three weeks before proceeding to endoscopy the patients had not received antimicrobial agents. The research was approved by the Ethics Committee of Shahid Beheshti University of Medical Sciences, Tehran, Iran (Code # 381). Two biopsy specimens were taken from the pyloric antrum in each patient. One of them was used for the rapid urease test and another biopsy was placed in sterile the range of 1.6-2 was considered as pure.Above and below this range were considered as contamination with protein and RNA, respectively. DNA was stored at -20°C for further studies.

**Detection of integrons**

The presence of class 1 and 2 integrons was investigated using PCR with degenerate primers described by Moura et al (11). Amplified fragments of the class 1 and 2 integrons were 280bp and 232bp, respectively which was performed in a GenAmp PCR system (Eppendorf, Hamburg, Germany) according to the following program: initial denaturation for 5 min at 94ºC, 35 cycles of denaturing at 94 ºC for 45 s, annealing at 58 ºC for 45 s, and extension at 72 ºC for1 min. The final extension was carried out at 72 ºC for 5 min.

**Statistical analysis**

Statistical analysis was performed using the SPSS version 18.0 software (SPSS Inc., Chicago, IL). Categorical variables were analyzed by Chi-square test. A *P *value ≤ 0.05 was statically significant.

## Results

Sixty five patients infected with *H. pylori *based on urea breath test and HpSA enrolled in this study, of whom 42 (64.6%) were male and 23 (35.4%) were female. The average age was 45 (median age 42±3.6 years, range 12 to 68 years). The age distribution was 6.2% for patients aged equal or less than 15 years, 18.5% for 16 to 45 years, 53.8% for 46 to 60 years, and 21.5% for equal or greater than 60 years. The present study demonstrates the highest frequency of *H. pylori *infection among 46-60 year age group (53.8%), while the age group less than 15 years had the lowest frequency (6.2%). Out of 65 patients participated in this study, 25 (38.5%) had peptic ulcer, 19 (29.2%) gastric cancer, 13 (20%) gastric ulcer and 8 (12.3%) gastric cancer with gastric ulcer.

**Antimicrobial susceptibility**

Regarding E-test, resistance rate was 73.8% for metronidazole (n=48), 43.1% for clarithromycin (n=28), 29.2% for tetracycline (n=19), 27.7% for amoxicillin (n=18), 23.1% for rifampicin (n=15) and 13.4% for levofloxacin (n=9). No significant association was observed between resistances to antibiotics and gender. Rates of resistance to metronidazole and tetracycline were higher in men (85.7% and 63.2%) compare to women (12.5% and 36.8); whereas more isolates were resistant to rifampicin, amoxicillin, levofloxacin, and clarithromycin were recovered from females.

Out of 48 isolates resistant to metronidazole, 23 (47.9%) of isolates had MIC > 8µg/ml, 12 (25%) had MIC ≥ 16µg/ml, 8 (16.7%) had MIC ≥ 32µg/ml and 5 (10.4%) had MIC ≥ 64µg/ ml. The MIC values for susceptible isolates to metronidazole (26.2%, n=17) was ranged from 0.25 to 4 µg/ml. Of 48 isolates resistant to metronidazole, 20 isolates were isolated from patients with peptic ulcer, 15 isolates from patients with gastric cancer, 5 isolates from patients with gastric ulcer and 8 isolates from patients with gastric cancer. The most of metronidazole resistant *H. pylori *patients were female. According to the results of the metronidazole E-test, all isolates were inhibited by metronidazole at similar MIC to clarithromycin by E-testing, which of them 18 (64.3%) of isolates had MIC ≥ 1 µg/ml and 10 (35.7%) had MIC ≥ 4 µg/ ml. Out of 28 isolates resistant to clarithromycin, 15 isolates were recovered from patients with gastric cancer, 12 isolates from patients with duodenal ulcer and one isolate from patients with gastric cancer and gastric ulcer. According to the EUCAST breakpoints, tetracycline resistance was found in 19 (29.2%) isolates and was inhibited at MIC ≥ 8 µg/ml. The MIC values of tetracycline for remaining 46 (70.8%) of isolates was ranged from 0. 25 to 1 µg/ml.

Among tetracycline-resistant isolates, 10 strains (52.6%) were isolated from patients with gastric cancer, five isolates (26.4%) from patients with peptic ulcer and 4 isolates (21%) from patients with gastric ulcer. Resistance to amoxicillin was observed in 18 isolates (27.7%) which five (27.8%) of isolates had MIC ≥ 2 µg/ml, 9 (50%) had MIC ≥ 4 µg/ml and four isolates (22.2%) had MIC ≥ 8 µg/ ml. Levofloxacin resistant isolates were found in patients with peptic ulcer (55.5%), and gastric cancer (44.5%). Increased resistance to rifampicin was observed in 15 (23.1%) of isolates. Out of 15 rifampicin-resistant isolates, nine (60%) of the isolates had MIC ≥ 2 µg/ml and six isolates (40%) had MIC ≥ 4 µg/ ml. The levofloxacin-resistant isolates were obtained from gastric cancer (73.3%), and peptic ulcer (26.7%) diseases. The lowest level of resistance was related to levofloxacin (13.4%). The results of levofloxacin MIC were as fallow: 25 (38.4%) had MIC 0.25 µg/ml, 28 (43.1%) had MIC 0. 5 µg/ml, three (4.6%) had MIC 1 µg/ml, four (6.2%) had MIC 2 µg/ml, three (4.6%) had MIC 4 µg/ml and two (3.1%) had MIC 8 µg/ml. Multidrug resistance (MDR) was defined as resistance to three or more antibiotics of different classes (12). Of 65 isolates tested in this study, 17 (26.1%) isolates were MDR. The predominant MDR profile among our isolates was included resistance to three (3.1%), four (4.6%), five (7.7%), and six (10.8%) antibiotics. Distribution of multiple antibiotic resistant patterns has been presented in Table 1.

The class 1 and 2 integrons were commonly found in eight (12.3%) and 15 (23.1%) isolates, respectively. The existence of class 3 integron and co-existence of class 1 and 2 integron were not confirmed in none of the isolates. The most isolates carrying class 2 integron (86.7%) were obtained from patients with gastric cancer and the remaining two isolates were obtained from patients with gastric ulcer (3.1%), while, all of the class 1 integron-bearing isolates were recovered from patients with peptic ulcer. The results of the existence of integrin’s and antimicrobial susceptibility testing of isolates have been shown in the Table 2.

## Discussion

The widespread emergence of MDR *H. pylori *is a serious and MIC_90_ 16 µg/ml. and increasing problem, which cause of eradication failure.

Out of 65 tested isolates, 28 isolates (43.1%) were resistant Effective therapy plays an important role in eradication of *H. pylori*-associated disorders and can increase the eradication rate to 85–90% (13).

**Table 1:** Frequency of antimicrobial resistance among *H. pylori* isolates

Resistance pattern	No (%)
MTZ	30(46.1)
AMX	1(1.5)
CLM	13(20)
MTZ+TET	4(6.2)
MTZ+AMX+LEV	2(3.1)
CLM+TET+AMX+RIF	3(4.6)
MTZ+CLM+TET+AMX+RIF	5(7.7)
MTZ+CLM+TET+AMX+RIF+LEV	7(10.8)
MTZ: Metronidazole; TET; Tetracycline; LEV; levofloxacin; CLM; clarithromycin; AMX; amoxicillin; RIF; rifampicin	

**Table 2:** Antibiotic resistance pattern and frequency of integron in *H.* *pylori* clinical isolates

**Antibiotics**	MIC(µg/ml)			Integron positive(n=23)		Inetgron negative(n=42)	
	50%	90%	R n (%)	S n (%)	R n (%)	S n (%)	R n (%)
metronidazole	2	4	48(73.8)	5 (21.7)	18(78.3)	12(28.6)	30(71.4)
clarithromycin	0.5	2	28(43.1)	8(34.8)	15(65.2)	29(69)	13(31)
tetracycline	1	1	19(29.2)	6(26.1)	17(73.9)	40(95.2)	2(4.8)
amoxicillin	0.5	1	18(27.7)	11(47.8)	12(52.2)	36(85.7)	6(14.3)
rifampicin	0.5	1	15(23.1)	13(56.5)	10(43.5)	37(88.1)	5(11.9)
levofloxacin	1	2	9(13.4)	20(87)	3(13)	36(85.7)	6(14.3)

Current treatment is primarily based on triple therapy with combinations of a proton-pump inhibitor, amoxicillin, metronidazole, and clarithromycin. Based on the literatures, antibiotic resistance of *H. pylori *isolates varies by region and country. Also, the rate of metronidazole resistance varies from 10% to 80% and is the main reason for chemotherapy failure. High rate of metronidazole resistance has hindered successful eradication of the infection (2, 6, 13).

High prevalence of metronidazole-resistant *H. pylori* isolates has been reported from Iran and some other Asian countries. The prevalence rate of metronidazole resistance between *H. pylori *strains is greatly variable (2, 14). The prevalence of *H. pylori *resistance to metronidazole in our study was relatively high (73.8%). This result was in agreement with our previous study in 2014 that the highest levels of resistance was related to metronidazole (66.3%) (14). In Milani’s study the rate of resistance to metronidazole in *H. pylori *isolates was found to be 76.8% (15). Similar to reports from other countries such as Columbia (82%) (16), Mexico (76.3%) (17), India (48.5%) (18) and Sweden (76%) (19) the rate of resistance to metronidazole in our isolates was high.

Although, metronidazole resistance is uncommon in developed countries, increased the rate of resistance to metronidazole has been reported recently in these countries (15). In a study that was performed on 2204 *H. pylori* clinical isolates in 18 European countries, during a 3-year period, the rate of resistance to metronidazole was found 34.9% (20). Chisholm et al. in a study in England and Wales, over a six- year period (2000-2005), showed that the rate of metronidazole resistance was varied from 28.6% to 36.3% (21). Another study conducted during a five-year period (1997-2001) in Korea, reported that the rate of resistance to metronidazole has increased from 25.2% in 1997 to 71.4% in 2000 (22). In a study conducted in Italy over a 13- year period from 1998 to 2012 on children with *H. pylori* infection, reduction in metronidazole resistance was reported from 56% in 1998/99 to 33% in 2011/12 (23). Based on different studies, there is difference in resistance rates to metronidazole which related to the frequent use of the drug, which is commonly prescribed for other diseases, especially parasitic conditions, and periodontal or gynaecological infections. In addition, use or abuse of this drug may contribute to the increased metronidazole resistance that seen in developing countries (5, 8, 20-22). However, role of various gene mutations in NADPH nitroreductase (*RdxA*), NADPH-flavin-oxidoreductase (*FrxA*), and ferredoxin likeenzymes (*FrxB*) in resistance to metronidazole should not be ignored (19). In line with our findings, O’Connor et al.

(24) showed that metronidazole resistance in females was more prevalent than males which may be because of the use of metronidazole for gynecological infections. Clarithromycin as key antibiotic in triple therapies for *H. pylori *infections have global increased resistance rates from 9% in 1998 to 17.6% in 2008 in Europe (25). In this study, the rate of resistance to clarithromycin was 43.1%, which is higher than other performed studies from India (11.8%) (18), Iran (18.4%) (14), and Brazil (19.5%) (26). This high prevalence of clarithromycin resistance may reflect inappropriate use of erythromycin in clinic, cross resistancewith other macrolides and point mutations.

Our results showed a considerable increased resistance rate to tetracycline (29.2%) in compared to previous study conducted in Iran (3.1%) (14), and other performed surveys in Korea (0.5%) (27), Bulgaria (1.9%) (12), and Turkey (0%) (28), India (16.2%) (18). Similarly high tetracycline resistance rates have been reported in Nigeria (29) that may be related to the low costs-associated overuse of this antibiotic, de novo mutations in the 16S rRNA, and acquisition of the mutant 16S rRNA gene through horizontal transfer.

The prevalence of amoxicillin-resistant *H. pylori *isolates was found to be high (27.7%). According to the literature, the resistance rate to amoxicillin varies from 0% in Turkey (28) to 17.6% in India (18). Reports of Iran also showed a wide discrepancy of amoxicillin resistance rates; for instance, in a study conducted by Siavoshi et al. in vitro resistance rates to amoxicillin was found 7.3% (30), while, Kohanteb et al. showed 20.8% resistance to amoxicillin in *H. pylori* strains. The overuse and misuse of antibiotic, point mutation on pbp1, and β-lactamase production may be key factors contributing to wide variation in amoxicillin resistance rates reported from different countries. We detected a high resistance rate to rifampicin (23.1%) in our study. Similarly, an Iranian study clarified that the prevalence of resistance to rifampicin in *H. pylori *isolates was 28.6% (15). In another study from Bulgaria (32), primary resistance against rifampicin was seen in 10.4–12% of the isolates. Moreover, it seems that the resistance to rifampicin is increasing that might be connected to the frequent use of this drug and point mutations affecting the *rpoB *gene.

It is well established that fluoroquinolones, particular levofloxacin and moxifloxacin are administered as an alternative to standard antibiotics against *H. pylori*. Like many other studies from Bulgaria (12), Japan (33), and Taiwan (34), rate of resistance to levofloxacin in present study was relatively low (13.4%).

Results of this study showed a 26.1% rate of MDR among *pylori *isolates. In same study, Milani et al. (15) reported a rate of 4% for MDR pattern. In this study, we detected that 13 isolates (20%) have double resistance to clarithromycin and metronidazole simultaneously which was higher than those in Turkey (45.4%) (28). Resistance to these antibiotics was reported earlier in America (29.3%), Europe (44.1%), Africa (92.4%), and Asia (18.9 %) (35).

In this survey, a dominant existence of class 2 integron (23.1%) was seen. This finding in agreement with a study from Argentina by Crespo et al. that revealed a high carrier rate (37.5%) of integron class 2 in clinical isolates of *H. pylori *(7). In a study conducted on metronidazole resistant *H. pylori *strains in China, authors reported that none of isolates carry different classes of integrin (36). This discrepancy in frequency of integron and gene cassettes in *H. pylori *isolates could be explained by overuse of antibiotics for treatment gastrointestinal disorders, various treatment protocols applied in different geographic regions and the ability of H pylori isolates in horizontal gene transfer among susceptible and resistant strains.

Given that relatively large number of *H. pylori *infected patients refer to private health care centers, evaluate the pattern of antibiotic resistance in *H. pylori *strains isolated from these patient is very important. In the present study, a high prevalence of antibiotic resistance in *H. pylori *isolates was reported from patients referred to private health care centers in Tehran, Iran. This dilemma emphasizes that guidelines for the management of *H. pylori *infection should be revised. We also confirmed the existence of class 2 integron with a relatively high frequency among our isolates. With regards to the role of integrons as a genetic element in horizontal transfer of antibiotic resistance and MDR, high frequency of integron in the present study may be associated with treatment failures in Iranian patients.
